# Functional genetics for all: engineered nucleases, CRISPR and the gene editing revolution

**DOI:** 10.1186/2041-9139-5-43

**Published:** 2014-11-18

**Authors:** Anna F Gilles, Michalis Averof

**Affiliations:** Institut de Génomique Fonctionnelle de Lyon (IGFL), École Normale Supérieure de Lyon, 46 Allée d’Italie, Lyon, 69364 France; BMIC graduate programme and Université Claude Bernard - Lyon 1, Lyon, France; Centre National de la Recherche Scientifique (CNRS), Lyon, France

**Keywords:** Comparative developmental biology, Model organisms, Gene targeting, Homologous recombination, Gene-editing nucleases, CRISPR

## Abstract

Developmental biology, as all experimental science, is empowered by technological advances. The availability of genetic tools in some species - designated as model organisms - has driven their use as major platforms for understanding development, physiology and behavior. Extending these tools to a wider range of species determines whether (and how) we can experimentally approach developmental diversity and evolution. During the last two decades, comparative developmental biology (evo-devo) was marked by the introduction of gene knockdown and deep sequencing technologies that are applicable to a wide range of species. These approaches allowed us to test the developmental role of specific genes in diverse species, to study biological processes that are not accessible in established models and, in some cases, to conduct genome-wide screens that overcome the limitations of the candidate gene approach. The recent discovery of CRISPR/Cas as a means of precise alterations into the genome promises to revolutionize developmental genetics. In this review we describe the development of gene editing tools, from zinc-finger nucleases to TALENs and CRISPR, and examine their application in gene targeting, their limitations and the opportunities they present for evo-devo. We outline their use in gene knock-out and knock-in approaches, and in manipulating gene functions by directing molecular effectors to specific sites in the genome. The ease-of-use and efficiency of CRISPR in diverse species provide an opportunity to close the technology gap that exists between established model organisms and emerging genetically-tractable species.

**Electronic supplementary material:**

The online version of this article (doi:10.1186/2041-9139-5-43) contains supplementary material, which is available to authorized users.

## Review

### Evo-devo: driven by technological advances

Our understanding of developmental mechanisms is shaped by the experimental models and approaches at hand. The powerful genetic approaches available in *Drosophila*, *C. elegans*, zebrafish, mice and *Arabidopsis* have largely driven developmental research during the past decades, focusing it on questions that are experimentally tractable in these species. However, biological diversity greatly surpasses what can be studied in these organisms. Phenomena such as regeneration, polyphenism and chromatin diminution challenge some of our conventional views of development, but are still poorly understood because they are not accessible in our current experimental models. Also, understanding the evolutionary paths by which diversity is generated requires that we compare developmental mechanisms among several animals, well beyond the established model organisms. Exploring these topics requires extending our genetic approaches to new species.

Establishing genetic tools in new organisms has always been a challenge for comparative developmental biology. Evo-devo started to flourish when cloning genes and studying their expression patterns in embryos could be extended to a wide range of animals, with the advent of PCR and whole-mount in situ hybridization techniques in the 1990s. These techniques allowed ‘candidate genes’ to be associated with specific developmental events in different animals and for evolutionary-developmental hypotheses to be formulated based on this information. Testing these hypotheses experimentally and exploring alternative possibilities in an unbiased fashion became, at that point, major challenges for the future of evo-devo.

Two important steps toward meeting those challenges were made since the late 1990s: the establishment of gene knockdown approaches based on RNAi and other antisense methods (see below) and the invention of low-cost deep sequencing technologies, which opened the door to unbiased genome-wide studies. Both methods could be applied to a wide range of species. We will focus here on functional genetics approaches.

The first important advance in this direction was made with the discovery of RNA interference (RNAi), a mechanism that uses small RNAs (processed from larger double-stranded precursors) to recognize and degrade specific RNA targets [1–4]. RNAi is a natural mechanism that evolved in eukaryotes to protect the genome against invading viruses and transposons [4]. This defense mechanism can be redirected to target specific mRNAs of interest by providing double-stranded RNA matching the target sequence. Since the RNAi machinery is found naturally in most eukaryotes, RNAi-mediated gene knockdown has turned out to be widely applicable. This approach has also been complemented by other antisense methods that target RNA using different types of oligonucleotides (morpholinos, antagomirs, LNAs and others [5–8]). Together, these antisense approaches have given us the opportunity to knock down gene functions at the RNA level in a wide range of animals, including cnidarians, arthropods, nematodes, planarians, annelids, echinoderms, tunicates and vertebrates (for example, [1, 3, 9–13]). RNAi-based screens in new experimental models have allowed us to study biological problems that were genetically intractable in the past, such as tissue homeostasis and regeneration in planarians [14] or particular aspects of insect physiology and development in beetles (http://ibeetle.uni-goettingen.de/).

The flurry of RNAi and other antisense studies carried out at the turn of the century revealed the power of these approaches, but also their limitations. Besides technical limitations relating to delivery, toxicity and off-target effects, for which solutions and appropriate controls can often be found [15–17], there are intrinsic limitations in the type of genetic manipulation that can be carried out: interference with gene function is usually transient, unlocalized, and primarily targets mRNA. Antisense approaches do not usually allow us to achieve complete loss of gene function, to perform stable genetic modification, to pursue gain-of-function and conditional approaches, or to study *cis-* regulatory elements.

In some organisms, these knockdown approaches have been complemented by transgenesis [18–24], which gives access to stable genetic modification and gain-of-function experiments via gene mis-expression. Transgenesis also enables the use of reporter constructs to study *cis-* regulatory elements and to generate tools for live imaging, as well as opportunities to generate mosaic animals, where clones of cells can be marked, genetically modified and compared to wild-type cells in the same individual [25]. The power of the transgenic approach in new experimental models can be seen, for example, in cell labeling and tracing experiments carried out to study regenerative progenitor cells in crustaceans and axolotls [26, 27]. The development of transgenesis requires a significant investment of time and effort, so this approach is still limited to few species.

Among the functional approaches that are applicable to a wide range of species, we can also count pharmacological treatments, which rely on the use of small molecule effectors to interfere with specific regulatory pathways [28].

Together, these technological advances allowed evo-devo to advance from descriptive cross-species comparisons (in the 1990s) to comparisons of gene function within a decade. In spite of this progress, most systems are still lagging far behind standard models in terms of experimental power and precision. The arrival of efficient and widely applicable gene editing approaches is set to narrow that gap, revolutionizing genetic approaches both in established models and in emerging experimental species.

### Gene targeting approaches

The ability to modify a chosen sequence in its native locus offers great advantages over conventional transgenesis and RNAi-mediated knockdown, both in terms of versatility and precision. It enables us to manipulate both coding sequences and *cis-* regulatory elements, to perform gain- and loss-of-function experiments, and to generate reporters and sensors that accurately reflect endogenous gene expression and function. Manipulating a gene in its native context is also a more precise approach because it allows us to study gene variants within their native *cis-* regulatory environment, where they are expressed in biologically meaningful levels and patterns.

Conventional gene targeting has exploited the natural ability of cells to recombine DNA fragments that bear homologous sequences, copying genetic changes from an engineered template sequence to a homologous site in the genome. In practice this often involves integrating an exogenous sequence, including appropriate markers, into the locus of interest. The efficiency of this process is low, in the order of 1 in 10^3^-10^7^ cells receiving the template DNA, and it occurs among a high background of non-homologous integration events [29, 30]. For this reason, conventional gene targeting is workable only in systems where we are able to screen very large numbers of transfected cells and select the rare targeting events, for example, in cultured mammalian cells and in yeast [29, 31–33].

The efficiency of gene targeting, however, is strongly enhanced (100 to 10,000-fold) when the targeted locus is disrupted by a double-strand DNA break [34–38]. For example, double-strand breaks produced by the excision of transposable elements are known to induce homologous recombination around the site of excision [39, 40]. Thus, to improve the efficiency and specificity of gene targeting, much attention has focused on directing double-strand breaks to unique DNA sequences in the genome.

**Figure 1:**
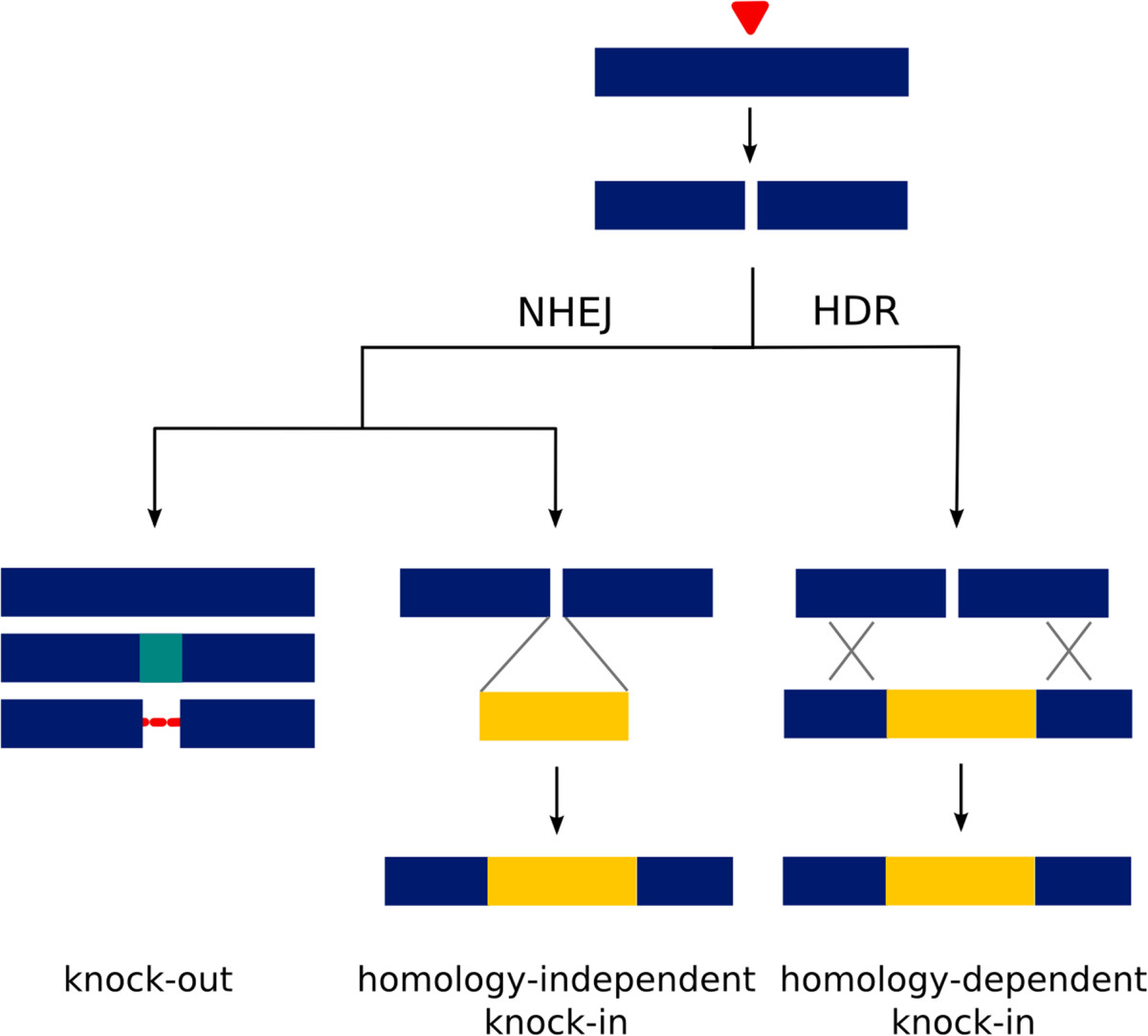
**Gene targeting strategies using targeted double-strand breaks.** When chromosomal DNA is cleaved (red arrowhead), the resulting double-strand break is repaired by non-homologous end joining (NHEJ) or by homology-dependent repair (HDR). NHEJ may result in perfect rejoining, of the ends, or in the introduction of point mutations and indels (knock-out). NHEJ may also join exogenous linear DNA (shown in yellow) to the broken ends of the chromosome (homology-independent knock-in); the orientation and reading frame in these insertions is random, unless directed by complementary overhangs [42, 44, 45]. HDR repairs the double-strand break by precise copying of a repair template carrying an exogenous sequence (shown in yellow) flanked by sequences with homology to the targeted locus (in blue) (homology-dependent knock-in). The repair template usually consists of circular plasmid DNA with long homology arms [46–50] or short single-stranded oligonucleotides (ssODNs) bearing 10 to 40 nucleotides of homologous sequence at each end [48, 50–52].

Double-strand breaks can elicit two types of molecular repair mechanism at the site of damage: non-homologous end joining (NHEJ) in which the broken ends are re-ligated to each other, or homology-directed repair (HDR) in which the break is repaired using a homologous DNA sequence as template (see [41]). NHEJ and HDR have different consequences, which are both relevant for gene editing. NHEJ is the predominant repair mechanism, but it is error-prone, resulting in the introduction of small insertions or deletions (indels) at the site of the break. Thus, NHEJ provides an efficient way to disrupt gene function (knock-out). In contrast, HDR is based on precise copying of the template and can serve to insert specific changes that have been engineered in the repair template (homology-dependent knock-in). NHEJ can also be used to ligate the broken ends to an exogenous linear DNA fragment, in the absence of sequence homology (homology-independent knock-in) [42–45]. NHEJ and HDR are almost ubiquitous in living organisms, so these targeting approaches (summarized in Figure 1) could in principle be applied in any species of interest.

One of the first approaches for generating double-strand breaks at specific sites in the genome exploited natural sequence-specific endonucleases with long recognition sequences. These so-called ‘meganucleases’ recognize sequences that are typically 15 to 30 nucleotides long, providing sufficient specificity to target unique sequences in eukaryotic genomes. Meganucleases have been successfully used in gene targeting [35, 36], but engineering these proteins to target new sequences has proven to be a major challenge [53]. Another approach has relied on artificial triple-helix-forming oligonucleotides [54, 55], but the scope of this approach is also limited because triple-helix formation is only possible with some sequences. Endonucleases with customizable sequence specificities have been a dream of gene targeting since the 1990s.

### Modular gene editing nucleases: zinc-finger nucleases and TALENs

A major breakthrough came with the realization that modular DNA recognition domains could be exploited combinatorially to generate nucleases targeting a wide range of sequences [56]. The zinc-finger domain, which typically recognizes 3-nucleotide motifs on DNA, was the first to be exploited in this way. Artificial enzymes, called zinc-finger nucleases (ZFNs), were engineered by joining several zinc-finger domains - recognizing adjacent trinucleotide motifs - to the catalytic domain of the endonuclease FokI (reviewed in [57]). Sequence-specificity was further increased by engineering ZFNs in a way that requires their heterodimerization through the FokI domain for efficient cleavage [58]. Thus, targeting an 18-nucleotide target site could (in principle) be achieved by a ZFN pair carrying 6 zinc-finger domains with the appropriate sequence specificities. To date, ZFNs have been used to target many genes in diverse organisms, exploiting both NHEJ-mediated knock-out and HDR-mediated knock-in approaches [57].

Despite their success in demonstrating the power of the modular approach, ZFNs suffer two major drawbacks that have limited their use. First, not all sequences can be targeted by ZFNs, because zinc-finger modules are not yet available for all possible nucleotide triplets (for example, [59]). Second, the sequence specificity of individual zinc-finger domains cannot always be precisely defined, and may be influenced by neighboring domains in the protein [60, 61]. This context dependence means that ZFN specificity is not easy to predict, leading to failures in targeting [62] and the need for costly design and testing.

The modular approach was taken a step further with the discovery of the TAL effector (TALE) DNA-binding modules of *Xanthomonas* bacteria, and their simple DNA recognition code [63, 64]. TAL proteins are transcription factors with a modular DNA-binding region that consists of multiple tandem repeats. Each of these repeats is a small DNA binding domain capable of recognizing a single nucleotide; two amino acid residues within each repeat determine its specificity for A, G, C or T, and this specificity is not significantly influenced by neighboring domains [65]. Thus, using the same combinatorial logic that was applied to ZFNs, TAL effector nuclease (TALEN) heterodimers with pre-defined sequence specificities can be generated by assembling multiple TAL repeats - one per nucleotide of the target site - linked to the catalytic domain of FokI [66–68]. Thus, a 24-nucleotide sequence can be targeted by generating a TALEN heterodimer, where each monomer consists of an array of 12 TAL repeats fused to FokI.

TALENs offer three great advantages over ZFNs. First, the modularity of the TAL domains and the simplicity of their DNA recognition code mean that virtually any sequence can be targeted by TALENs. Second, the specificity of TAL domains does not appear to be as context-dependent as that of zinc fingers, which results in more accurate predictions of target specificity and a higher targeting success rate. Third, gene targeting experiments in diverse species reveal that TALENs are very efficient, yielding targeting efficiencies as high as 30 to 100% for NHEJ-mediated knock-outs and 1 to 10% for HDR-mediated knock-ins (measured as the fraction of injected individuals giving rise to progeny carrying a targeted allele). High targeting rates have been achieved in a wide range of organisms, including insects, nematodes, annelids, tunicates, vertebrates and diverse plants [69–76].

The fact that each TAL domain targets a single nucleotide means that long TAL arrays need to be assembled in order to target unique sequences in a eukaryotic genome. Ingenious protocols have been developed for this purpose [77–79], bringing TALEN technology within the reach of every competent molecular biology lab.

### Simple and efficient gene editing using the RNA-guided nuclease CRISPR/Cas9

The last two years have seen the development of a new approach to build endonucleases with customized sequence specificities, which has revolutionized gene editing by its simplicity and efficiency. The approach is borrowed from a highly efficient immune mechanism of bacteria and archaea, which employs RNA-guided endonucleases to specifically target and degrade viral DNA [80–82] (reviewed in [83]).

The genomes of many prokaryotes possess hypervariable loci, termed clustered regularly interspaced short palindromic repeats (CRISPR), which incorporate short sequences from invading viruses and express them in the form of CRISPR-derived RNAs (crRNAs). These small RNAs associate with specific CRISPR-associated (Cas) proteins to form an active CRISPR/Cas endonuclease complex, whose specificity is determined by simple base complementarity between crRNA and the target viral DNA. Immunity to a viral infection is determined by the presence of corresponding viral sequences in CRISPR loci [80, 84, 85]. The CRISPR mechanism bears some striking analogies with eukaryotic RNAi and piRNA-mediated defense mechanisms^a^[83, 86].

**Figure 2:**
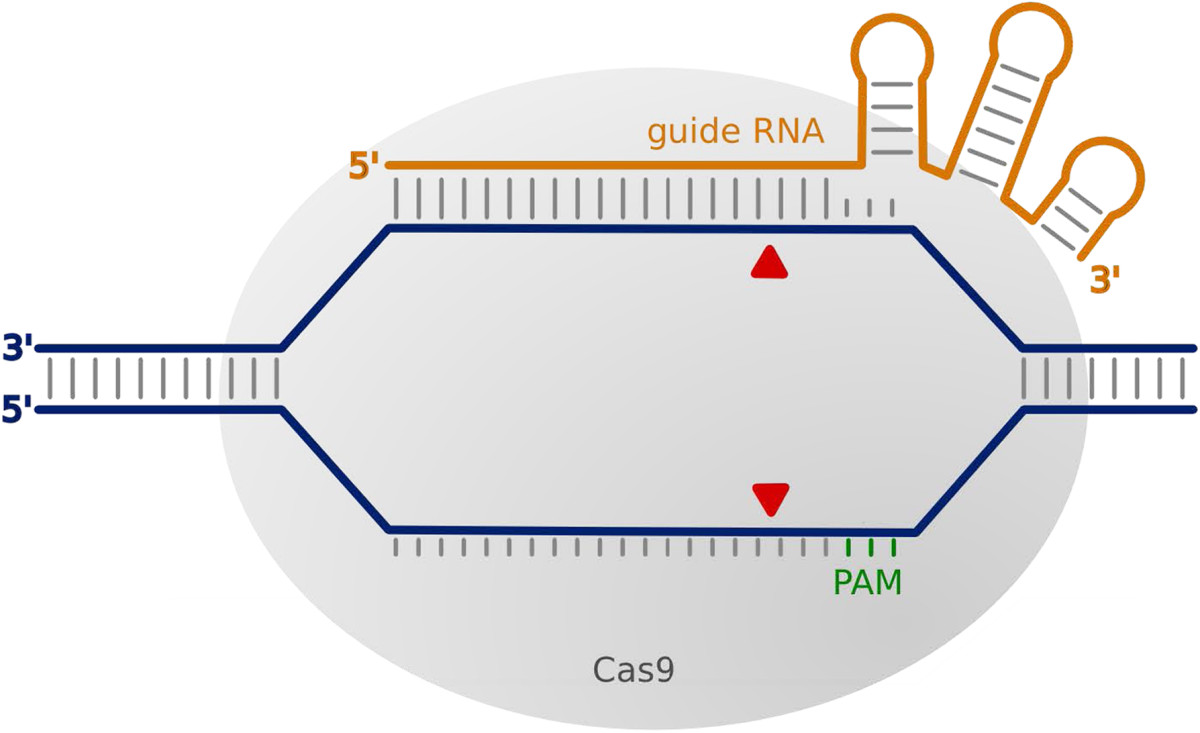
**CRISPR/Cas9 interacting with target DNA.** The CRISPR/Cas9 complex of *Streptococcus pyogenes* consists of the Cas9 protein (in gray) and a guide RNA that is a chimera of natural crRNA and tracRNA (in orange). The targeting sequence at the 5′end of the guide RNA base-pairs with complementary sequences on the target DNA (in blue); the targeting sequence is 20 nucleotides long, but may be shortened to increase specificity [88] (the addition of 1 to 2 unpaired nucleotides at the 5′ end is also tolerated [51, 88]). The presence of a PAM (protospacer adjacent motif, NGG for *Streptococcus pyogenes*), located immediately downstream of the 20-nucleotide sequence targeted by the guide RNA, is also essential for target recognition and cleavage. The PAM sequence does not have a counterpart on the guide RNA. Following recognition of the PAM and base-pairing between the guide RNA and the target, Cas9 cleaves each of the target DNA strands a few nucleotides upstream of the PAM (red arrowheads). Each strand is cleaved by a different nuclease domain present in Cas9 (HNH and RuvC domains). These domains have been mutated independently to generate Cas9 nickases [82, 87].

In a ground-breaking study published in 2012, Jinek and colleagues demonstrated that this nucleotide-based recognition mechanism could provide a straightforward approach for generating customizable nucleases for gene targeting [87]. They used the CRISPR system of *Streptococcus pyogenes*, which involves a single Cas protein (Cas9) and two RNAs (crRNA and trans-acting antisense RNA, also known as tracRNA) to build an active CRISPR/Cas endonuclease complex. Jinek *et al*. showed that it is possible to combine these two RNAs into a single chimeric guide RNA (known as gRNA or sgRNA) that can efficiently direct Cas9 activity to specific DNA targets *in vitro* (Figure 2). The guide RNA has a region of 20 nucleotides at its 5′ end, which binds the target DNA and determines specificity; any 20-nucleotide sequence (N_20_) can be placed at that site^b^. The 3′ region of the guide RNA, corresponding to the bacterial tracRNA, is an invariable sequence that is required to form a complex with Cas9.

Target recognition also depends on additional interactions between Cas9 and the target DNA, which require the presence of a specific sequence motif, the ‘protospacer adjacent motif’ (PAM), immediately downstream of the 20-nucleotide sequence targeted by the guide RNA. The PAM sequence does not have a counterpart on the guide RNA (Figure 2). *Streptococcus pyogenes* CRISPR/Cas9 requires a PAM that is NGG; it can thus target any sequence that matches the motif N_20_NGG. Once a target is bound, two separate nuclease domains of Cas9 are involved in cleaving each strand of the target DNA. Cleavage occurs within the guide RNA target region, usually three nucleotides upstream of the PAM [81, 82, 87].

Within less than two years since the first demonstration of CRISPR-mediated gene editing [89–93], there has been an explosion of reports describing the application of CRISPR in diverse animal and plant species (reviewed in [94]). The approaches to generate knock-outs and knock-ins are similar to those previously described for ZFNs and TALENs, relying on the endonuclease to generate a double-strand break at the targeted locus and on the cell’s imprecise or template-directed mechanisms of repairing that break (Figure 1). However, compared to ZFNs and TALENs, CRISPR has radically improved the accessibility of gene targeting due to its straightforward approach for customizing sequence specificity, via target-specific guide RNAs. Its targeting efficiencies are comparable with the best efficiencies achieved using TALENs in a wide range of animals and plants (for example, [46, 51, 95, 96]), including organisms where gene targeting is not yet widely available, such as silkmoths, axolotls, Xenopus and monkeys [97–100]. Moreover, while TALEN activity is inhibited by DNA methylation [101], CRISPR activity does not appear to be so [102]. Table 1 summarizes the relative benefits and drawbacks of ZFNs, TALENs and CRISPR.

**Table 1:** **Comparison of ZFN, TALEN and CRISPR approaches**

	ZFN	TALEN	CRISPR
**Mode of action**	DNA break targeted by protein-DNA recognition	DNA break targeted by protein-DNA recognition	DNA break targeted by RNA-DNA base complementarity
**Nuclease design and assembly**	Difficult (commercial services expensive)	Feasible in most labs but labor intensive	Easy (see Table 2)
**Success rate of nuclease design** ^**a**^	Low	High	High
**Targeting efficiency**	Variable	High with most nucleases	High with most guide RNAs
**Target range**	Limited by range and context-dependence of ZF modules	Unlimited	Limited by PAM sequence (potentially unlimited)
**Potential off-target effects**	Yes	Yes	Yes
**Sensitivity to DNA methylation**	Not known	Sensitive to CpG methylation	Not sensitive to CpG methylation
**High throughput targeting**	No	Limited	Yes

^a^see [71, 103]. PAM, protospacer adjacent motif; TALEN, TAL effector nuclease; ZFN, zinc-finger nuclease.

### CRISPR delivery and target range

Different approaches have been used to deliver gene-editing nucleases into target cells, including microinjection and transfection. CRISPR systems require the delivery of Cas9 together with guide RNA. Cas9 may be expressed from a helper plasmid carrying the coding sequence of Cas9 (fused with a nuclear localization signal and sometimes ‘codon-optimized’) under the control of an appropriate promoter. Alternatively, if a promoter is unavailable, Cas9 can be provided in the form of *in vitro* transcribed capped mRNA or as purified recombinant protein [104, 105]. In established models, such as *Drosophila*, transgenic strains have been generated that express Cas9 in the germ line [47, 48, 96].

Delivery of the guide RNA is more constrained because *in vitro* transcription or plasmid-derived expression impose some limitations on the sequence of the RNA and may, therefore, influence the range of potential targets. *In vitro* transcription of RNA is usually carried out using the RNA polymerase of bacteriophages T7, T3 or SP6, which generate transcripts that start with GG (T3 or T7 RNA polymerase) or GA (SP6 polymerase) [106]. The alternative to *in vitro* synthesis is to express the guide RNA from a plasmid or transgene *in vivo*. Small RNAs are conventionally expressed using RNA polymerase III promoters, because they often require precisely defined initiation and termination sites and should not enter the mRNA processing pathway. Thus, guide RNAs are usually expressed via the U6 snRNA promoter [46, 89–91, 107]; U6 promoters generate transcripts that start with a G.

In principle, these sequence constraints dictate that, using *Streptococcus pyogenes* Cas9 (with NGG as a PAM), we can optimally target sequences that contain GGN_18_NGG or GAN_18_NGG motifs using *in vitro* transcribed guide RNAs and GN_19_NGG using the U6 promoter to drive guide RNA expression. In practice, however, it seems that mismatches at the 5′ end of the guide RNA are well tolerated, giving acceptable levels of gene targeting [51, 88]. Moreover, alternative guide RNA expression strategies are emerging, which overcome the constraints imposed by the U6 promoter [108–110]. Thus, the PAM sequence may be the only stringent limitation to CRISPR’s target range.

PAM recognition seems to play a key role in CRISPR target recognition [111], so the requirement for a PAM in the target sequence is likely to remain. The sequence constraints imposed by the PAM may be overcome by exploiting the natural diversity of CRISPR systems [112–114], or by rational design and artificial selection of Cas9 variants that recognize different PAM sequences.

### Off-target effects

Whether using ZFNs, TALENs or CRISPR, targeting a chosen, unique sequence in the genome may be accompanied by unintended cleavage at other loci. Several studies have investigated the specificity and potential off-targets of CRISPR [89, 102, 115–121], focusing on the stringency of base-pairing between the guide RNA and the target. These studies have established that mismatches are tolerated, especially at the 5′ end of the guide RNA, but that there are no simple rules predicting the likelihood of mis-targeting based on the number and position of mismatches. In some cases, even sequences with multiple mismatches to the guide RNA were targeted efficiently [116]. In one study, targeting specificity deteriorated when high concentrations of CRISPR/Cas9 were used [118].

A recent study has also highlighted the role played by the PAM sequence as CRISPR/Cas9 interrogates complex DNA sequences to identify target sites [111]. The study shows that the CRISPR/Cas9 complex first identifies potential targets based on the PAM sequence, and then interrogates these for sequence complementarity with the guide RNA. The complex does not appear to interact with sequences that match the guide RNA targeting motif (N_20_) but have no adjacent PAM. These observations suggest that off-targets will generally not include sequences that are lacking the PAM. In species where the genome sequence is known, computational tools are now routinely used to select guide RNAs and to evaluate potential off-targeting based on sequence similarity and on the presence of a PAM (see Online Resources for CRISPR in Table 2).

**Table 2:** **A quick guide to CRISPR for beginners** ^**a**^

*1.*	*Prerequisites*
	• Delivery method, reaching the germline or other cells of interest: microinjection, transfection, electroporation
	• Genomic sequence of target genes
	• Robust phenotypic assays to determine the effect of gene targeting
*2.*	*Experimental strategy*
	• Decide on the targeting approach (knock-in or knock-out), depending on whether you want to disrupt gene function, engineer a specific mutation, generate a reporter, *etcetera*.
	• When testing CRISPR for the first time, choose a simple knock-out approach, selecting targets that produce phenotypes that are easy to score, such as pigmentation genes or a GFP transgene [70, 98, 122], or genes with a known, robust and specific phenotype.
	*For knock-ins*
	• To knock-in large constructs, use HDR templates in which the knock-in construct is flanked by homology arms - typically >1 kb in length - matching the sequences on either side of the double-strand break [46–49]; shorter homology arms give lower efficiencies [50]. Provide the template as a circular plasmid.
	• To introduce small changes, use synthetic single-stranded oligos (ssODNs) bearing 10 to 40 nucleotides of homologous sequence at each end as templates for HDR [48, 50–52].
	• The sequence targeted by CRISPR should be mutated in the repair template to protect the template and targeted alleles from cleavage.
	• Alternatively, a homology-independent knock-in approach (see Figure 1) may be used to knock-in large DNA fragments [42, 45] or short double-stranded oligos (dsODNs) [44]. Using this approach, the insertion may be imprecise [45] or directed by complementary overhangs [42, 44].
	• Select an approach that will minimize lethality due to NHEJ-mediated indels in somatic tissues, for example, by restricting CRISPR/Cas activity to the germline [48, 123], targeting constructs to introns, or adopting a strategy that improves the relative efficiency of knock-ins [42, 45, 50].
*3.*	*Design of guide RNAs - finding target sequences*
	• Use the most reliable genomic sequence available for the target gene. Consider that the targeted site may bear nucleotide polymorphisms; if this is likely to be an issue, obtain sequences from the strain used for gene targeting and/or test multiple guide RNAs.
	• Use online software to search for potential target sites (see Online Resources for CRISPR, below). The software search a given sequence for sites with a suitable PAM motif (NGG for *S. pyogenes* Cas9) and additional sequence constraints depending on the mode of guide RNA production (GGN_18_NGG for *in vitro* T7-synthesis of guide RNAs, GN_19_NGG for U6-mediated expression). The latter requirements can be relaxed, as extra Gs may be added to the 5′ end of the guide RNA without significantly compromising targeting efficiency [51, 88].
	• When working with a sequenced genome, the software can also detect potential unintended targets and help select guide RNAs with fewer off-targets.
	• Although the presence of the PAM sequence at the genomic target site is essential, it should not be included in the guide RNA (see Figure 2). For an N_20_NGG target site, only the N_20_ sequence is incorporated at the 5′end of the guide RNA.
	• Design and test multiple guide RNAs, if possible, to control for off-target effects and because some guide RNAs fail (due to polymorphisms, RNA secondary structure or for unexpected reasons).
	• Strategies to reduce off-target effects may require special design of guide RNAs: paired nickase approaches require pairs of target sequences offset by no more that 30 nucleotides on opposite DNA strands [44, 109, 117, 119, 124, 125]; truncated guide RNAs bear targeting sequences that are shorter than 20 nucleotides [88].
*4.*	*Providing guide RNAs and Cas9*
	• Guide RNAs are easily generated by cloning pairs of synthetic oligos, corresponding to the two strands of the target sequence (determined above), into vectors carrying the invariable portion of the guide RNA (available at http://www.addgene.org/CRISPR). Cloning is facilitated by a restriction site on the vector - usually BbsI or BsaI, which does not constrain the cloned sequence - and compatible overhangs in the annealed oligos.
	• The guide RNAs can be expressed either by *in vitro* transcription via the bacteriophage T3, T7 or SP6 promoters, or by *in vivo* expression via the U6 promoter. For initial experiments in species where U6 promoters and terminators are untested, choose *in vitro* synthesis of the guide RNA. Vectors and protocols can be found at http://www.addgene.org/CRISPR.
	• Cas9 can be expressed from a helper plasmid carrying the coding sequence of Cas9 under the control of an appropriate promoter. Alternatively, if a promoter is unavailable for the species of interest, Cas9 can be provided in the form of *in vitro* transcribed capped mRNA or as purified recombinant protein [104, 105]. For initial experiments performed by microinjection, the use of recombinant Cas9 protein overcomes uncertainties with untested promoters and mRNA translation.
*5.*	*Rapid assays of CRISPR activity and genotyping*
	• The melting curve and surveyor or T7E1 endonuclease assays are invaluable for a rapid assessment of CRISPR activity in new species, for routine testing of new guide RNAs prior to more laborious experiments, and for genotyping animals at specific target sites. These assays detect indels and other point mutations generated by NHEJ. They rely on PCR and require only a small amount of starting material.
	• Genomic DNA is extracted from embryos or tissues to be tested and PCR is performed using primers that flank the target site. Untreated genomic DNA gives a PCR product with perfectly annealed strands (unless there are natural polymorphisms within the fragment), whereas mutagenized genomic DNA also yields some heteroduplex DNA, consisting of strands that differ by small indels and point mutations. The following assays are used to detect of these mismatches.
	- Surveyor/T7E1 endonuclease assays are based on cleavage of the heteroduplexes by a mismatch-specific endonuclease - either Surveyor or T7 endonuclease 1 [126, 127]. Cleavage products, indicating the presence of mispaired DNA, are detected by electrophoresis on an agarose gel. This is a sensitive detection method, best performed on 400 to 800 bp amplicons with target sites positioned near the middle.
	- The melting curve assay [128] relies on the fact that heteroduplex DNA has a lower melting temperature than the corresponding homoduplex fragments. That temperature difference, which is in the order of 1 to 2°C for 100 to 200 bp fragments, can be detected by performing melting curves in real-time PCR instruments with high temperature resolution.
	• More specific PCR-based assays can be devised for knock-in approaches, employing pairs of primers that span the genomic locus and knock-in fragment.
	• The PCR products can be cloned and sequenced to examine the nature and spectrum of corresponding mutations.
*6.*	*Scoring phenotypes*
	• The effects of CRISPR targeting can be assessed in the animals where CRISPR was delivered (G0) or in their progeny. It is important to keep in mind that G0s are mosaics where only some cells are likely to carry alleles targeted by CRISPR; in the best cases a significant proportion of the animal shows bi-allelic targeting and a corresponding phenotype. The degree and distribution of targeted cell clones however are difficult to determine, unless a cell-autonomous marker is used (for example, targeting of some pigment genes, knock-in of GFP).
	• If the germline of G0s has been hit, targeted alleles will be recovered in the next generation (G1). In contrast to G0s, G1 individuals are non-mosaic and may inherit one targeted allele (per locus) from the CRISPR-targeted parent. Animals may be genotyped by PCR (see above) and crossed to produce homozygotes and to maintain mutant lines.
	• Choosing reliable, specific phenotypic assays and appropriate controls is crucial. Phenotypes may be subtle or show incomplete penetrance.
*7.*	*Off-target effects and controls*
	• Unintended targets (off-targets) may be anywhere in the genome and are difficult to predict. Two strategies can help to overcome problems with off-target effects: appropriate experimental design allowing us to detect and account for off-target effects and approaches that improve the specificity of CRISPR.
	• In most cases it is possible to control for off-targets by using different guide RNAs to achieve targeting; guide RNAs targeting different sequences are very unlikely to share the same off-targets. Alleles generated using different guide RNAs may be brought together by crossing, in heteroallelic combinations that are likely to complement off-target mutations.
	• The specificity of CRISPR can be significantly improved by using paired nickases [44, 109, 117, 119, 124, 125] or truncated guide RNAs [88] (see main text).
	• Off-target mutations will segregate away from targeted alleles in genetic crosses, unless they are linked on the chromosome.
*8.*	*Online resources for CRISPR*
	• General
	http://www.genome-engineering.org/crispr
	http://www.addgene.org/CRISPR
	http://www.flycrispr.molbio.wisc.edu
	http://www.crisprflydesign.org
	http://groups.google.com/forum/#!forum/crispr
	• Software for designing guide RNAs
	http://crispr.mit.edu
	http://www.addgene.org/CRISPR/reference/#gRNA
	http://tools.flycrispr.molbio.wisc.edu/targetFinder
	http://www.e-crisp.org/E-CRISP/designcrispr.html
	http://www.rgenome.net/cas-offinder
	• CRISPR technology is recent and rapidly evolving. Online resources are likely to change, as improvements and new tools are introduced.

^a^Useful practical advice and a protocol (applied to cell lines) can also be found in [129].

A number of approaches can be taken to confront the off-target problem and to mitigate its effects. First, it is often possible to control for unspecific effects through appropriate experimental design. A strategy commonly employed in RNAi studies is to examine whether consistent phenotypes are obtained by targeting different parts of a gene, using non-overlapping double-stranded RNA fragments or siRNAs [17]. The same strategy can be easily applied in most cases of gene targeting by CRISPR, by using different guide RNAs. Guide RNAs targeting different sequences are very unlikely to share the same off-target effects. Notably, heteroallelic combinations of knock-outs generated using different guide RNAs are likely to complement off-target mutations and to give highly specific knock-out phenotypes.

Strategies for improving the specificity of CRISPR are also beginning to emerge, exploiting the combined action of pairs of CRISPR nucleases, or methods that increase the specificity of individual nucleases. The first approach relies on mutants of Cas9, known as nickases, that cleave only one of the two DNA strands. Using such mutants, a double strand break can be generated by targeting a pair of closely linked single-strand breaks (nicks) on opposite DNA strands. The requirement that these nicks coincide drastically improves targeting specificity compared to that of single CRISPR nucleases [44, 117, 119, 124]. A variant of this approach combines the CRISPR/Cas9 DNA binding activity with the FokI endonuclease, whose dimerization requirements ensure that no nicking occurs at off-target sites [109, 125]. For efficient cleavage, these ‘paired nickase’ approaches require that adjacent target sites, offset by up to 30 nucleotides, can be found on opposite DNA strands.

A second approach to increase the specificity of targeting by CRISPR relies on the observation that short recognition sequences are less forgiving in terms of allowed mismatches between the guide RNA and its targets [88] (similar observations on specificity and target size have been made with TALENs, [65]). Thus, guide RNAs with targeting sequences of 17 to 19 nucleotides show high targeting efficiencies and much reduced off-target effects compared to ones with canonical 20-nucleotide targeting sequences [88].

Ultimately, it may also be possible to improve the stringency of CRISPR target recognition by selecting or engineering Cas9 nucleases that are intrinsically less promiscuous.

## Opportunities for evo-devo and future challenges

CRISPR-mediated gene targeting opens a wide range of opportunities, both in established model organisms and in newly emerging ones. A quick guide for applying CRISPR in new species is given in Table 2.

Knock-out approaches will surely be more widely applied in newly established experimental systems due to the extraordinary efficiency of NHEJ-mediated knock-out, which approaches 100% in some species. Generating a null-allele or disrupting a specific *cis-* regulatory element now seems within reach in a wide range of animals and will be primarily limited by our ability to screen for these mutations (by phenotype or by PCR) and to maintain mutant stocks.

To some extent, the high efficiency of CRISPR- and TALEN-mediated knock-out may also help to overcome the problem of stock-keeping. The high frequency of bi-allelic knock-out in injected embryos using CRISPR or TALEN has raised the possibility of carrying out ‘G0 genetics’, examining phenotypes directly in the injected embryos (for example, [48, 98, 122, 130, 131]). Phenotypic analysis without crosses could be used as a ‘quick and dirty’ approach in species that have long generation times, or for preliminary screens on a large number of candidate genes, similar to RNAi. The obvious drawback of this type of analysis is the genetic mosaicism of the organism, which is difficult to control and will lead to partial and variable phenotypes.

However, mosaicism could also be an advantage in contexts where genetic manipulation within specific cell lineages or in random cell clones is desirable to overcome lethality, or to assess cell autonomous versus non-autonomous effects of gene function. Particularly so when the extent of mosaicism can be monitored or manipulated (see [48]). Tissue- and stage-specific knock-outs may be achieved by manipulating the expression of Cas9 [48].

Knock-in approaches provide an even wider range of opportunities, including precise modification of genes in their native genomic context, and generating visible reporters for regulatory and physiological events, and drivers for transgene expression. The major challenge to overcome here is that, for any given guide RNA or TALEN, the frequency of mutagenic NHEJ repair will be much higher (by an order of magnitude) than the frequency of HDR- or NHEJ-driven knock-in (see Figure 1). A high knock-out frequency in the somatic cells of injected animals can lead to lethality that prevents knock-ins to be recovered in the next generation. This problem could be overcome in a number of ways: by using germline-specific cis-regulatory elements to restrict the activity of Cas9 to the germline [48, 123]; by targeting sites that are unlikely to be lethal if mutated, for example, targeting knock-ins to intronic sequences, with appropriate splice signals to generate functional gene fusions; by finding ways to improve the efficiency of HDR relative to NHEJ, such as by knocking down the activity of DNA ligase 4 or other factors that are essential for NHEJ [49, 50, 132]; or by developing strategies that exploit NHEJ-mediated gene knock-ins to achieve higher efficiencies [42–45] (see Figure 1).

Beyond knock-out and knock-in strategies, the ability to direct double-strand breaks to specific sites in the genome raises the prospect of chromosome engineering. Generating chromosomal deletions and inversions is presently not one of the usual tools employed in evo-devo, but one only needs to consider the enormous contribution of balancer chromosomes in *Drosophila* genetics to appreciate its potential value in emerging model organisms [133]. Balancers are invaluable tools for selecting and keeping track of chromosomes bearing mutations, especially when these mutations are deleterious and not associated with a dominant marker (for example, the knock-out of an essential gene). CRISPR and TALENs now allow us to generate targeted chromosomal inversions [134–136] associated with recessive lethal mutations and, thus, to create balancers, in organisms where a genome sequence and map are available.

Last but not least, the customizable specificity of CRISPR may be harnessed to direct other molecular effectors - besides nucleases - to specific sites in the genome (reviewed in [137]). For example, catalytically inactive versions of Cas9 have been used to interfere with transcription [138, 139], coupled with transcriptional activators, repressors or chromatin modifiers to generate artificial transcriptional regulators [117, 139–143], or linked with fluorescent proteins to reveal chromosome dynamics [144]. This approach allows us to manipulate the activity of regulatory elements in their native context without introducing changes in their nucleotide sequence, providing tools of unprecedented precision in our efforts to manipulate and to understand gene regulation.

CRISPR technology is young - barely two years old - and still rapidly evolving. Improvements, new applications and adaptations of the technique to new species have been published at overwhelming speed during the last year, and surely more are forthcoming. This is a true revolution for comparative studies. Practical issues, such as the delivery method and our ability to select and to propagate mutants, are still likely to limit the full deployment of CRISPR in many species. Notwithstanding these issues, targeted mutagenesis and precise gene editing are now within reach in a very wide range of organisms.

### Endnotes

^a^It is interesting to note that the adaptive diversification, specificity and efficiency of immunity mechanisms provide the basis for some of the most powerful tools in molecular biology: restriction enzymes, antibodies, RNAi and CRISPR.

^b^Somewhat shorter sequences may also be used (see Off-target effects).

## Authors’ original submitted files for images

Below are the links to the authors’ original submitted files for images. Authors’ original file for figure 1 Authors’ original file for figure 2 Authors’ original file for figure 3

## Abbreviations

Cas: CRISPR-associated protein
CRISPR: Clustered regularly interspaced short palindromic repeats
crRNA: CRISPR-derived RNA
HDR: Homology-directed repair
NHEJ: Non-homologous end joining
PAM: Protospacer adjacent motif
RNAi: RNA Interference
TALEN: TAL effector nuclease
tracRNA: Trans-acting antisense RNA
ZFN: Zinc-finger nuclease.
