# Supplementary figures and images for: Functional genetics for all: engineered nucleases, CRISPR and the gene editing revolution
Source: EvoDevo. 2014 Nov 18;5:43. doi: 10.1186/2041-9139-5-43 (PMC4332929; doi:10.1186/2041-9139-5-43)

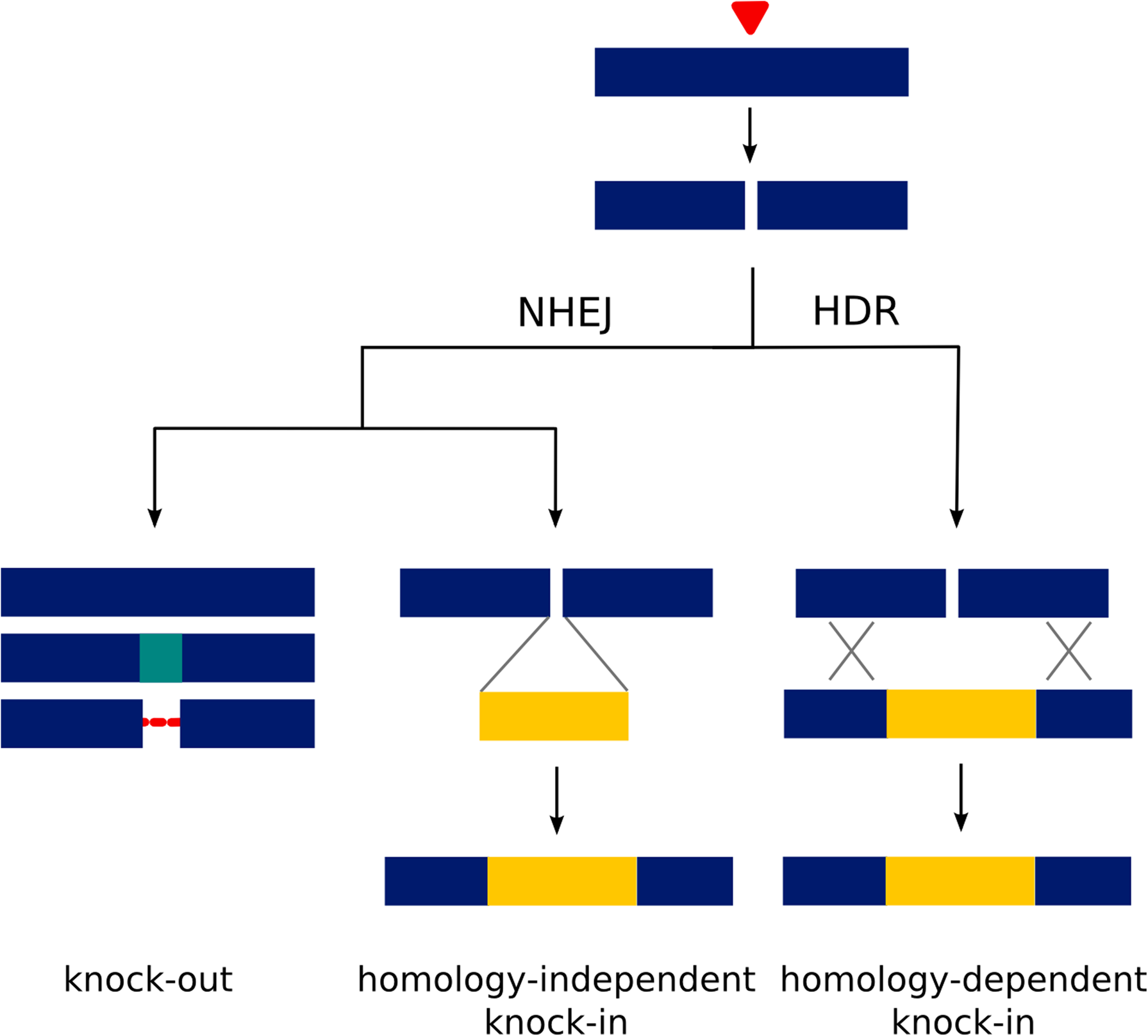

Supplement: Supplementary file 1 — Authors’ original file for figure 1 [file 13227_2014_130_MOESM1_ESM.tiff]

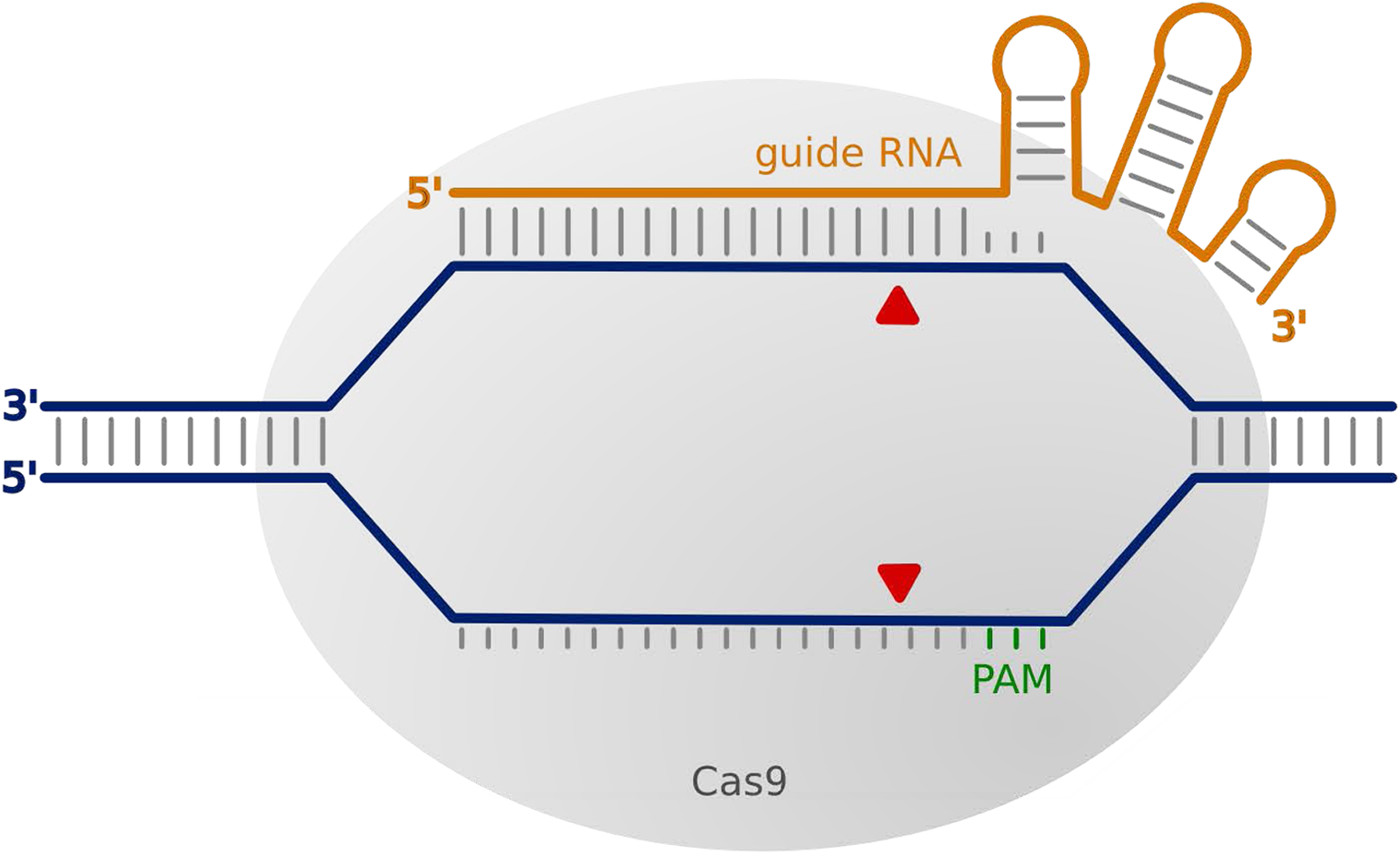

Supplement: Supplementary file 2 — Authors’ original file for figure 2 [file 13227_2014_130_MOESM2_ESM.tif]

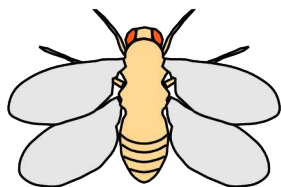

mutagenesis

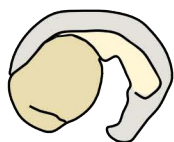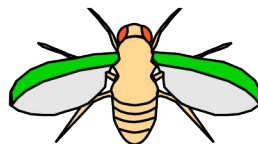

knock-in

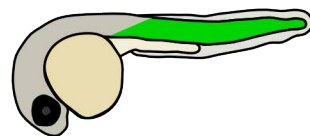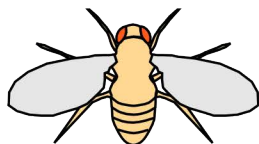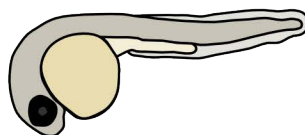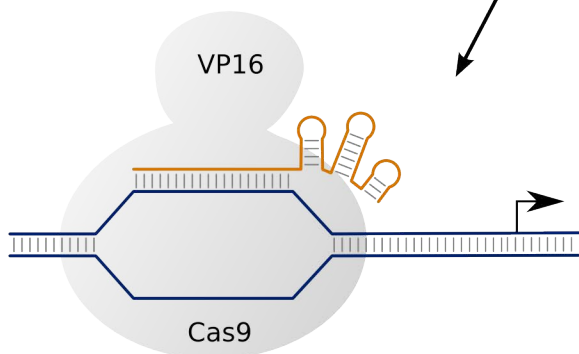

artificial gene regulators

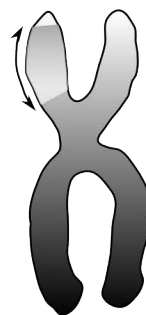

chromosomal rearrangements

Supplement: Supplementary file 3 — Authors’ original file for figure 3 [file 13227_2014_130_MOESM3_ESM.pdf]
